# Transparent and Flexible Capacitors with an Ultrathin Structure by Using Graphene as Bottom Electrodes

**DOI:** 10.3390/nano7120418

**Published:** 2017-11-28

**Authors:** Tao Guo, Guozhen Zhang, Xi Su, Heng Zhang, Jiaxian Wan, Xue Chen, Hao Wu, Chang Liu

**Affiliations:** 1 Key Laboratory of Artificial Micro- and Nano-Structures of Ministry of Education, and School of Physics and Technology, Wuhan University, Wuhan 430072, China; tao.g@whu.edu.cn (T.G.); guozhen.zhang@whu.edu.cn (G.Z.); xsu@whu.edu.cn (X.S.); zhheng@whu.edu.cn (H.Z.); j.x.wan@whu.edu.cn (J.W.); xuechen@whu.edu.cn (X.C.); 2 Hubei Nuclear Solid Physics Key Laboratory, and School of Physics and Technology, Wuhan University, Wuhan 430072, China

**Keywords:** transparent and flexible capacitors, graphene, ZrO_2_ films, atomic layer deposition

## Abstract

Ultrathin, transparent and flexible capacitors using graphene as the bottom electrodes were directly fabricated on polyethylene naphthalate (PEN) substrates. ZrO_2_ dielectric films were deposited on the treated surface of graphene by atomic layer deposition (ALD). The deposition process did not introduce any detectible defects in the graphene, as indicated by Raman measurements, guaranteeing the electrical performances of the graphene electrodes. The Aluminum-doped zinc oxide (AZO) films were prepared as the top electrodes using the ALD technique. The capacitors presented a high capacitance density (10.3 fF/μm^2^ at 10 kHz) and a relatively low leakage current (5.3 × 10^−6^ A/cm^2^ at 1 V). Bending tests revealed that the capacitors were able to work normally at an outward bending radius of 10 mm without any deterioration of electrical properties. The capacitors exhibited an average optical transmittance of close to 70% at visible wavelengths. Thus, it opens the door to practical applications in transparent integrated circuits.

## 1. Introduction

In recent years, transparent and flexible flat-panel displays formed on plastic substrates have attracted substantial attention, particularly in the field of wearable electronics, owing to their fascinating advantages, which include high optical transparency, good bendability, their light weight and their low cost [1,2]. In the pixel-drive circuit of displays, capacitors usually play the role of charging and discharging at very high speed [3,4]. A high capacitance is needed to ensure the color brightness of each pixel, while a low leakage current means a prolonged charge storage time when the pixel is in the off state [5]. Apart from this, bendability and transparency should also be seriously considered when the capacitors are applied in flexible electronics.

To achieve an excellent transparency and flexibility of the capacitors, the selection of high-performance electrode materials is crucial. Compared to ordinary transparent conducting films, graphene has superior performances in transmittance, conductivity and mechanical strength, owing to its thickness of one carbon atom [6,7]. As is well known, the tensile or compressive stress on the film surface is proportional to the film thickness. Along this line, the entire capacitor structure can be made ultrathin when graphene is used as the electrode, which facilitates substantial improvements in flexibility. Furthermore, a single layer of graphene has a very high transmittance of 97.7% in the visible region, which ensures the high transparency of the capacitors [8]. At present, commercial large-area graphene can be conveniently prepared by chemical vapor deposition (CVD) and transferred onto various target substrates. The lowest reported sheet resistance (~125 Ω/□) of the CVD-graphene is comparable to that of industrialized indium-tin oxide (ITO) electrodes (~100 Ω/□) [8,9]. Thus, graphene is very suitable for fabricating transparent and flexible capacitors.

Capacitors with high capacitance, small featured size and low power consumption can be realized by employing high-k materials with wide bandgaps as dielectrics. Many high-k materials, including Al_2_O_3_ [10], TiO_2_ [11], HfO_2_ [12], ZrO_2_ [13], have been thoroughly investigated in the last few years. Among them, ZrO_2_ demonstrated great potential due to its high relative permittivity of about 25, and its relatively large bandgap of about 5.6 eV [14]. In addition, its excellent temperature stability and low permeation rates for ambient gases are attractive in the sense that they can improve device reliability [15]. In our recent work, capacitors using ZrO_2_ film as the dielectric layer showed an ultralow leakage current while achieving a high capacitance density [16].

In order to fabricate graphene-based electronics, high-quality dielectric films on the top of the graphene are required as electrostatic gate dielectrics or tunnel barriers [17,18]. For the purpose of providing accurate control over the thickness of dielectric films, dielectric layers should be made in nanoscale and be of uniform coverage on the graphene, without pinholes. Fortunately, atomic layer deposition (ALD) is a powerful method for preparing high-quality dielectric films. It adopts an inherent self-limiting growth mode to realize the ALD process and demonstrates many advantages, such as accurate thickness control, high uniformity over a large area, and low defect density [19]. Although the preparation of Al_2_O_3_ and HfO_2_ on graphene has been studied [20,21,22,23], the reports referring to ALD-grown ZrO_2_ dielectric films are quite few.

In this work, ultrathin, transparent and flexible capacitors using graphene for the bottom electrodes were fabricated directly on polyethylene naphthalate (PEN) substrates. A two-step ALD process at temperatures of 100 and 150 °C was developed to gain high-quality ZrO_2_ films. Al doped ZnO (AZO) films were then deposited in-situ on the ZrO_2_ surface as the top electrodes. The capacitors had a total thickness of under 165 nm, and presented a high capacitance density with a low leakage current. Bending tests indicated that the capacitors can work at an outward bending radius of 10 mm without any deterioration of the performance.

## 2. Results and Discussion

The surface of graphene is sp^2^-hybridized and lacks dangling bonds, which are necessary for chemical reactions during the initial nucleation of the ALD process. Generally, it is hard to grow high-quality dielectric films on the top of graphene. To solve this problem, functionalization of the graphene surface is performed to meet the demand of nucleation sites. So far, researchers have developed various surface-modification methods, which can be divided into chemical and physical methods [24,25,26]. However, these methods may damage the graphene, and inevitably introduce defects into the graphene. To overcome the drawbacks, Zheng et al. [27] recently reported enhanced nucleation of Al_2_O_3_ using adsorption of H_2_O on the graphene surface. In this way, a low leakage current was obtained by a two-step process at temperatures of 100 and 200 °C. Practical application for photoelectric devices, however, was not mentioned.

The surface of graphene is chemically inert and lacks dangling bonds, yet H_2_O molecules can be physically absorbed by the van der Waals forces. Absorbed H_2_O molecules could act as nucleation sites, and their consistency would play an important role in the growth of the ZrO_2_ seed layers. The final state of water mainly depends on the chamber temperature and the H_2_O dosage. The essence of H_2_O physical adsorption is related to water vapor liquefying on the surface of graphene. This happens easily when the temperature of the H_2_O vapor reaches its liquefaction point [27,28]. Thus, the initial temperature of the ALD chamber was set at 100 °C to ensure H_2_O molecules were able to continuously adhere to the graphene by gas–solid physical absorption. H_2_O dosage can be controlled by the pulse time of liquid water. Prior to the deposition of the ZrO_2_ dielectric films, liquid water was introduced into the ALD chamber by the carrier gas N_2_. The liquid water pulse time and the interval between two pulses were set at the same period, 10 s, which gave enough time for the H_2_O molecules to be transformed between the two phases (liquid and gas) and absorbed onto the graphene. In order to find out the optimum H_2_O treatment conditions, and to obtain continuous nucleation sites for the ZrO_2_ seed layers, 1 to 6 cycles of H_2_O treatment were tested. After that, 5 nm thick ZrO_2_ seed layers were grown at 100 °C with 42 ALD cycles.

**Figure 1:**
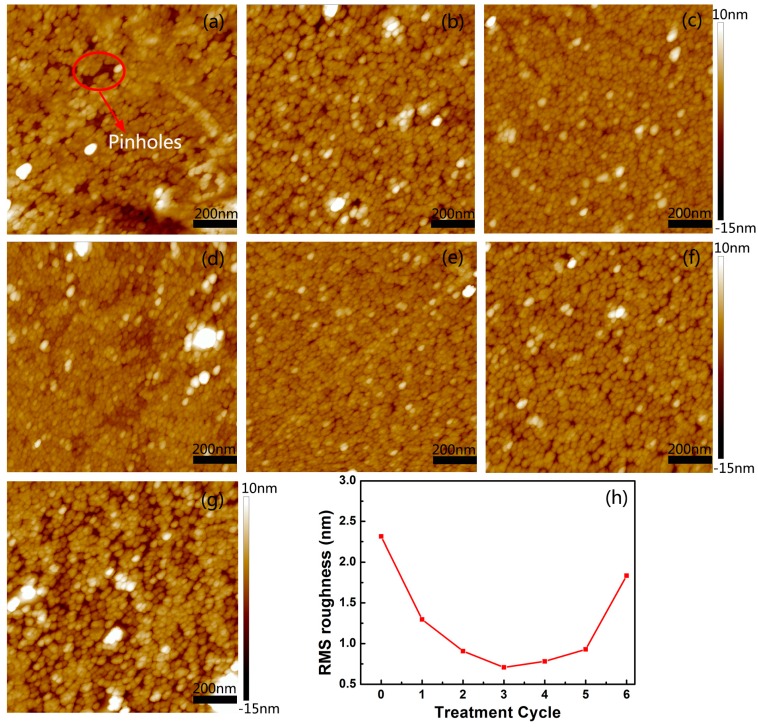
AFM images of ZrO_2_ seed layers on graphene with different cycles of H_2_O treatment at 100 °C: (**a**) 0; (**b**) 1; (**c**) 2; (**d**) 3; (**e**) 4; (**f**) 5; and (**g**) 6 cycles; (**h**) The RMS roughness of the ZrO_2_ surface.

Figure 1 presents the results of atomic force microscope (AFM) measurements. The surface morphologies of the ZrO_2_ seed layers on graphene with H_2_O treatment for 0–6 cycles are shown in Figure 1a–g, respectively. From Figure 1a,b, one can see that many pinholes exist in the ZrO_2_ film with H_2_O treatment for 0 and 1 cycle. When increasing the number of H_2_O treatment cycles, the pinholes reduced obviously, and the ZrO_2_ films became denser, as seen in Figure 1c,d. For H_2_O treatment for 3 cycles, the pinholes could hardly be seen in the seed layer. However, when number of H_2_O treatment cycles reached 4, the pinholes recurred, as can be seen in Figure 1e. Furthermore, their density increased with increasing H_2_O treatment cycles, as seen in Figure 1f,g. Meanwhile, the root-mean-square (RMS) roughness of the ZrO_2_ seed layers was minimized after H_2_O treatment for 3 cycles, as seen in Figure 1h. Fewer H_2_O treatment cycles (<3) led to insufficient nucleation sites, causing a relatively rough surface. However, when it was over-treated by H_2_O (treatment cycles ≥ 4), lots of clusters were formed on the graphene surface due to the van der Waals force between H_2_O molecules, and the graphene was unable to overcome the inter-molecular attraction and surface tension of the H_2_O molecules, resulting in a substantial increase in the surface roughness, and poor morphology in the subsequent ALD-grown ZrO_2_ seed layers. Thus, the optimum number of cycles of H_2_O treatment was determined to be 3 cycles. 5 nm thick ZrO_2_ seed layers were then grown under this optimum condition at 100 °C. Subsequently, higher-quality ZrO_2_ dielectric films were grown at 150 °C.

**Figure 2:**
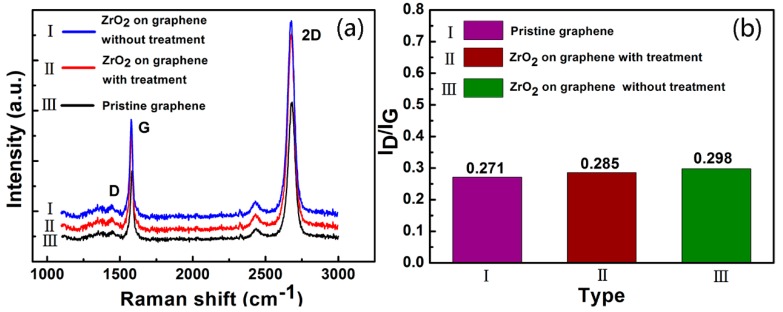
(**a**) Raman spectra and (**b**) the ratio of I_D_/I_G_ of pristine graphene and graphene covered with 5 nm thick ZrO_2_ films with and without surface treatment.

High-quality graphene is crucial for electronic applications. Raman spectroscopy measurements were used to qualitatively evaluate whether the H_2_O treatment and subsequent ALD-growth of the ZrO_2_ films introduced significant defects, or even deteriorated the structural and electrical properties of graphene. Figure 2a shows three typical Raman peaks (D, G, 2D) of the pristine graphene and graphene covered by the ZrO_2_ films with and without the surface treatment [29]. Pristine graphene presents distinct G and 2D peaks at ~1600 and ~2700 cm^−1^, respectively. The D peak could be observed in the pristine graphene at 1350 cm^−1^, which was related to the defects in graphene, and indicated that the defects of the CVD-grown pristine graphene could not be ignored [30]. The ratio of the peak intensities between D and G (I_D_/I_G_) clearly reflected the defect density of graphene [31]. Figure 2b shows the I_D_/I_G_ ratios, which were almost unchanged with and without surface treatment compared to that of the pristine graphene, implying that the process of surface treatment and subsequent ALD growth of the ZrO_2_ films at 100 °C did not introduce noticeable defects into the graphene. As a consequence, no significant defects were generated in the graphene, not only in the process of H_2_O treatment, but also in the process of ALD growth of the ZrO_2_ films.

**Figure 3:**
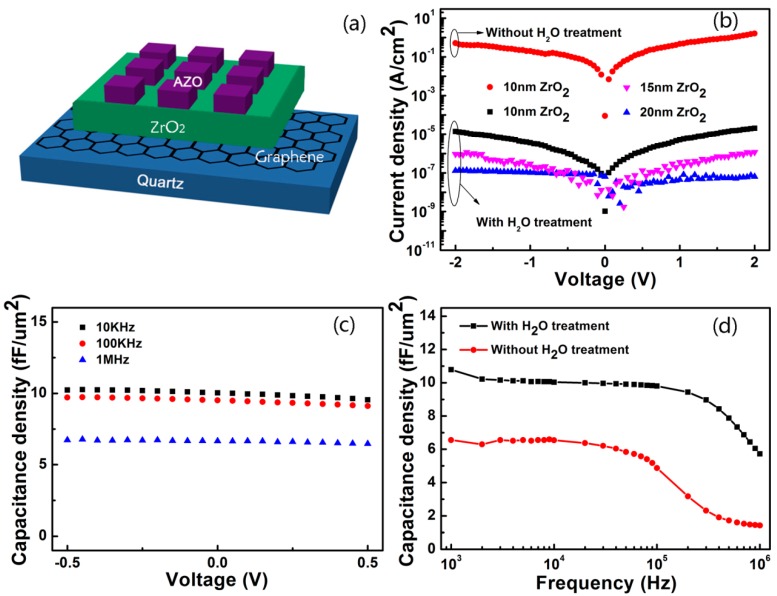
(**a**) A schematic diagram of the capacitors on quartz substrates; the electrical characteristics of the capacitors; (**b**) I-V characteristics of the capacitors with different ZrO_2_ film thicknesses with and without the surface treatment; (**c**) C-V characteristics of the capacitors at different frequencies between 10 kHz and 1 MHz; (**d**) C-F characteristics of the capacitors between 1 kHz and 10 MHz.

Figure 3a shows the schematic diagram of the capacitors fabricated on quartz substrates. I-V curves were measured to analyze the dependence of the leakage current on the thickness of the dielectric layers prepared at 150 °C with and without the surface treatment, as shown in Figure 3b. It should be noted that the leakage current of the capacitors was very high without the surface treatment when the thickness of the ZrO_2_ dielectric films was 10 nm. In contrast, the leakage current decreased to 5.3 × 10^−6^ A/cm^2^ after the surface treatment. However, when the thickness of ZrO_2_ dielectric films was increased to 15 and 20 nm, the leakage currents decreased to 3.6 × 10^−7^ (15 nm) and even 4.5 × 10^−8^ (20 nm) A/cm^2^ at 1 V, indicating that nano-capacitors with low leakage currents can be prepared on graphene whose surface has been physically treated by H_2_O adsorption. To balance the leakage current and capacitance of the capacitors, the thickness of the ZrO_2_ dielectric films was finally fixed at 10 nm in this study.

As shown in Figure 3c, the capacitance density reached 10.3 fF/μm^2^ at 10 kHz, which corresponds to a dielectric constant of 11.6. The capacitance density declined slightly when the voltage was swept from −0.5 to 0.5 V. A possible reason relates to the residuals of the incomplete chemical reaction between TDMAZ and H_2_O at the relatively low temperatures of 100 and 150 °C, which formed ZrO_2_ films that contained impurities. When the ZrO_2_ films were grown at 200 °C, the chemical reaction was more sufficient, and the capacitance remained almost unchanged in a voltage range from −2 to 2 V, as seen before in our previous work [32]. In this study, however, we used the PEN substrates to prepare flexible capacitors that cannot bear heating at 200 °C. Figure 3d presents a comparison of the capacitance density between those with and without the surface treatment by H_2_O. The former remained nearly unchanged throughout the frequency range from 1 to 100 kHz, and declined significantly at frequencies above 100 kHz. The dielectric loss occurring at high frequencies originated mainly from the relatively high sheet resistance of the graphene (~500 Ω/□) and AZO (~400 Ω/□) electrodes. The latter had pretty smaller capacitance densities, which dropped slightly when the frequency was above 10 kHz, but dropped heavily above 100 kHz. Thus, it can be concluded that the capacitors can work steadily at frequencies between 1 and 100 kHz with H_2_O treatment of the graphene.

**Figure 4:**
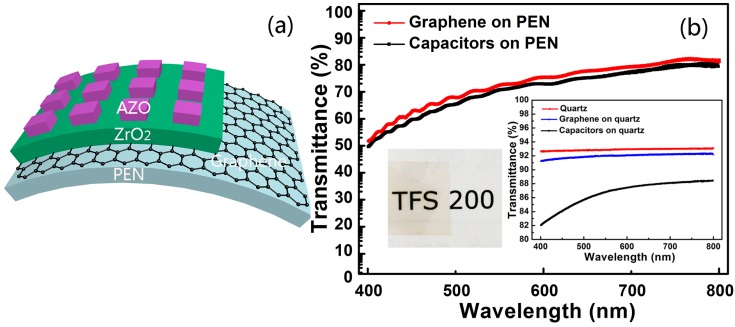
(**a**) The schematic diagram of the ultrathin, transparent and flexible capacitors; (**b**) The optical transmittance spectra of the capacitors on PEN substrates. The inset shows the optical photograph of the actual capacitor device with the characters “TFS 200” in the background, and the optical transmittance spectra of graphene and capacitors on quartz substrates.

In order for them to be used in flexible capacitors, the flexibility properties of the graphene electrodes need to be investigated. Figure 4a shows the schematic diagram of the ultrathin, transparent and flexible capacitors fabricated directly on PEN substrate. Figure 4b shows the transmittance spectra of the capacitors in the wavelength range from 400 to 800 nm. The capacitors exhibited an average optical transmittance of close to 70%. The loss of transmittance originated mostly from the PEN substrates, as seen in Figure 4b. The inset in Figure 4b shows that the graphene had an average transmittance of over 90%, and the capacitors on quartz substrates exhibited a transmittance of over 80% throughout the whole visible range. The English characters “TFS” can be clearly seen through the transparent capacitor, as shown in the inset of Figure 4b. Therefore, the intrinsic transmittance of our flexible nano-capacitors is valuable for applications in transparent electronics.

**Figure 5:**
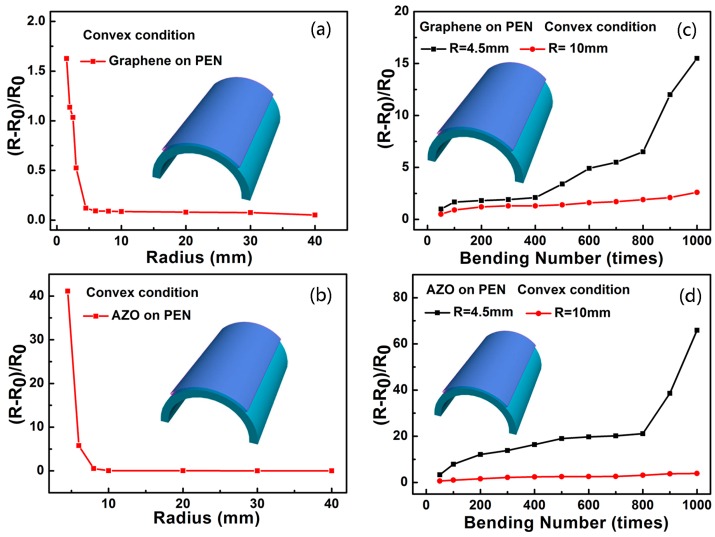
Variation of sheet resistance of (**a**) graphene and (**b**) AZO in convex conditions with different bending radii. Variation of sheet resistance of (**c**) graphene and (**d**) AZO in convex conditions with different bending numbers at a radius of 4.5 and 10 mm, respectively.

The flexibility and anti-fatigue properties of the graphene and 150 nm thick AZO electrodes on PEN were studied by bending tests in convex condition. The variation in sheet resistance is expressed as (R − R_0_)/R_0_, where R_0_ and R represent the sheet resistance before and after bending, respectively. Figure 5a shows that the sheet resistance of the graphene electrodes stayed nearly invariant when the bending radius was decreased from 40 to 4.5 mm, but increased apparently below 3 mm. Meanwhile, the sheet resistance of the AZO electrodes remained almost unchanged in a bending radius range from 40 to 10 mm, as shown in Figure 5b. The anti-fatigue properties of the graphene and AZO electrodes were tested at bending radii of 4.5 and 10 mm, and the data are shown in Figure 5c,d, respectively. The sheet resistance of the graphene electrodes increased slightly with lower numbers of bending times (≤400), and rose significantly above 400 times at a radius of 4.5 mm. In contrast, the electrical properties of the AZO electrodes deteriorated seriously. Fortunately, the sheet resistance of graphene and AZO electrodes remained almost unchanged below 800 bending times, and increased only a little when it reached 1000 times at a radius of 10 mm. As a consequence, the graphene and AZO electrodes show excellent flexibility and relatively good anti-fatigue properties at larger bending radius (≥10 mm). It should be pointed out that the graphene electrodes were easily damaged, and their resistance increased significantly during the bending process in the concave condition, which deteriorated the flexibility of the capacitors. Therefore, bending tests are crucial for those capacitors that use graphene and AZO as the electrodes.

**Figure 6:**
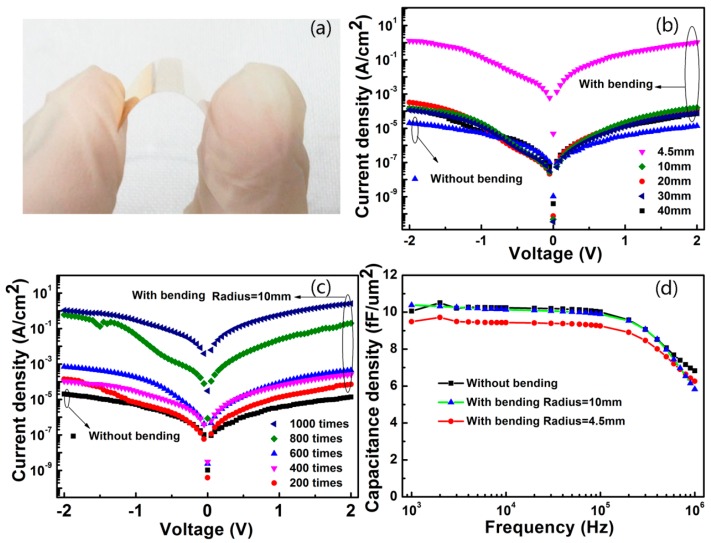
(**a**) The image of the capacitors on PEN; electrical characteristics of the capacitors in bending conditions; (**b**) I-V curves at different bending radii; (**c**) I-V curves with different bending times at a radius of 10 mm; (**d**) C-F curves with and without bending.

For the capacitors on PEN substrates without bending, the electrical characteristics remained almost the same as those on quartz substrates. Figure 6a shows the image of the capacitors in bending conditions. In Figure 6b, the leakage current of the flexible capacitors did not change markedly after bending in a radius range larger than 10 mm when the voltage was swept between −2 and 2 V, but differed greatly at a bending radius of 4.5 mm. The leakage current increased from 3.3 × 10^−6^ to 7.9 × 10^−4^ (A/cm^2^) at 1 V when the number of bending times fell into the range between 0 and 600 times, and increased greatly to 6.8 × 10^−2^ (A/cm^2^) at around 800 times and even 0.6 (A/cm^2^) at around 1000 times at a radius of 10 mm, as shown in Figure 6c. In Figure 6d, the capacitance of the capacitors remained almost invariant at a bending radius of 10 mm, but dropped slightly at a bending radius of 4.5 mm, relative to that without bending in a frequency range between 1 kHz and 1 MHz. Therefore, the flexible capacitors are able to work in harsh bending conditions even at a radius of 4.5 mm. Of course, the larger the bending radius, the better the anti-fatigue properties. These results were superior to what we have reported before [32].

**Figure 7:**
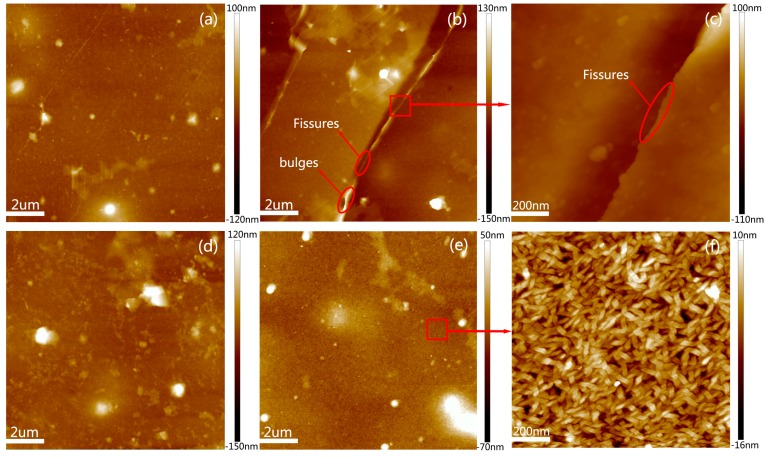
The AFM images of the devices after bending and cyclic bending 1000 times at a radius of 10 mm. The surface of ZrO_2_ dielectric layers (**a**) without and (**b**) with cyclic bending; (**c**) magnified image. The surface of AZO electrodes (**d**) without and (**e**) with cyclic bending; (**f**) magnified image.

The surface morphologies of the devices were studied by AFM measurements to understand why the leakage current increased significantly and the capacitance density dropped slightly after the bending tests. The surface morphologies of the ZrO_2_ dielectric layers after bending and cyclic bending are shown in Figure 7a–c. No obvious flaws appeared in the ZrO_2_ dielectric layer after bending at a radius of 10 mm, as seen in Figure 7a. However, after cyclic bending for 1000 times, as can be seen from Figure 7b, fissures and bulges were apparent. Figure 7c shows the magnified fissures that provide a transport channel for current between the graphene and AZO electrodes. There is no doubt that the transport channel would be increasingly unobstructed for the current with an increase in the bending number, which results in a substantial increase in the leakage current. The capacitance is expressed as C = (ε_0_ε_r_s_v_)/d, where C represents the capacitance density, and ε_0_, ε_r_, s_v_, and d represent the vacuum dielectric constant, the relative dielectric constant, the longitudinal area of the polar plate, and the thickness of dielectric layer, respectively. The s_v_ might decrease and the d increase due to the appearance of the fissures and bulges in the ZrO_2_ dielectric layer after the bending tests. Meanwhile, ε_0_ and ε_r_ remain unchanged. Hence, the capacitance density slightly drops after the bending tests. The fissures and bulges did not exist in the AZO electrodes after bending and cyclic bending 1000 times, as shown in Figure 7d,e, respectively. The details of the surface morphologies can be seen more clearly in the magnified image, which are presented in Figure 7f. The small tapered crystals of AZO films can be observed, and the film surface is smooth and continuous. This can be accounted for by the smaller sizes of the AZO pads (100 × 100 μm^2^) in comparison to the bending radius (10 mm). Therefore, the AZO electrodes sustained almost no damage in the bending process.

**Figure 8:**
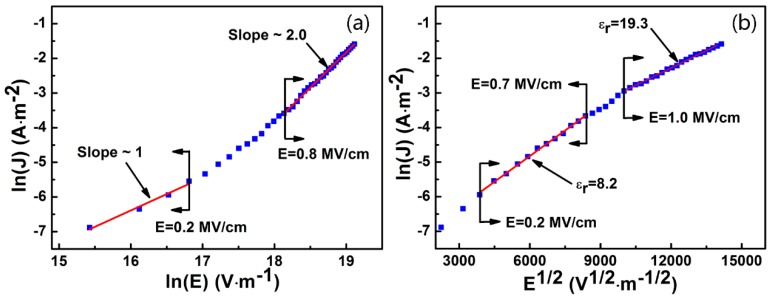
The leakage current mechanism: (**a**) Ohmic plots, space-charge-limited-current (SCLC) plots and (**b**) Schottky plots.

To understand how the leakage current of the flexible capacitors is generated, models of Ohmic conduction, space-charge-limited-current (SCLC) and Schottky emission were used. Generally, Schottky emission and Ohmic conduction play an important role in the mechanism of the leakage current at low fields. Figure 8a shows the relationship between ln(J) and ln(E). Here, the J and E represent the leakage current density and the electric field, respectively. The slope was close to 1 when the field was below 0.2 MV/cm, which demonstrated that Ohmic conduction existed. Such a behavior disappeared when the field was above 0.2 MV/cm. The slope was about 2 at higher fields (E > 0.8 MV/cm), which satisfied the SCLC model. In Figure 8b, a linear relationship was observed for the Schottky emission, with ln(J) proportional to E^1/2^. As the fitted relative dielectric constant (8.2) approached the measured one (11.6), the Schottky emission dominated at low fields (0.2 < E < 0.7 MV/cm). However, the fitted relative dielectric constant reached 19.3 at high fields (E > 1 MV/cm), which differed greatly from the measured one (11.6). Thus, the Schottky emission could be excluded at high fields. It can be concluded that Ohmic conduction dominates when the field is lower than 0.2 MV/cm, while the Schottky emission plays the main role when 0.2 < E < 0.7 MV/cm, and the SCLC mechanism is suitable when E > 0.8 MV/cm.

## 3. Experimental Section

### 3.1. Device Fabrication Methods

Before device fabrication, PEN and quartz substrates were ultrasonically cleaned in acetone for 10 min, followed by heating in alcohol at 60 °C for 10 min and in deionized water for 10 min, and then the cleaned substrates were purged by high-pressure N_2_ to remove the remaining water and other particles from the surfaces. Commercial single-layer graphene (SG) was grown by CVD on copper foil (2D Carbon Tech Inc. LTD, Changzhou, China). SG was then transferred onto the PEN and quartz substrates from Cu foil by using the roll-to-roll method [8]. Subsequently, the substrates with graphene were rapidly transferred to the ALD (TFS-200, Beneq, Vantaa, Helsinki, Finland) chamber and H_2_O molecules were distributed uniformly on the graphene by gas-solid physical absorption at 100 °C. Thereafter, 5 nm thick ZrO_2_ films were deposited on the graphene at 100 °C as seed layers. Then, thicker ZrO_2_ dielectric films with varying thicknesses were deposited on the ZrO_2_ seed layers at 150 °C. Finally, 150 nm thick AZO films were deposited in situ on the ZrO_2_ dielectric films at 150 °C as the top electrodes. Photolithography and wet etching processes were carried out to define the as-prepared top AZO films into isolated pads. The final AZO pads were about 100 × 100 μm^2^ in area, and the distance between two pads was about 400 μm.

### 3.2. Growth Conditions

Tetrakisdimethylamino zirconium (TDMAZ) and H_2_O were used to grow ZrO_2_ at 100 and 150 °C. The growth rate was about 0.2 nm/cycle at 100 °C and 0.1 nm/cycle at 150 °C, respectively. Diethyl zinc (DEZn), trimethyl aluminum (TMA) and H_2_O were used to deposit the AZO films, and the growth rate was approximately 4 nm per period. Each period consisted of 20 cycles of ZnO and 1 cycle of Al_2_O_3_. The growth rates of ZnO and Al_2_O_3_ at 150 °C were 0.2 and 0.1 nm/cycle, respectively.

### 3.3. Characterization Methods

The surface morphology of the ZrO_2_ films was measured by atomic force microscope (AFM, Bruker Multimode 8, Karisruhe, Germany). Raman spectroscopy (Raman, HORIBA Jobin Yvon LabRam, Paris, France) was implemented to determine whether the treatment of the graphene surface is suitable for the ALD process. The sheet resistance of the graphene and AZO electrodes was measured by hall measurements (Model 7707A, Lake Shore, WA, USA). Capacitance versus frequency (C-F), capacitance versus voltage (C-V), and leakage current versus voltage (I-V) were measured with a Keithley 4200 semiconductor analyzer (Keithley, Johnston, OH, USA). The optical transmittance was measured by a UV-VIS-NIR spectrophotometer (Varian Cary 5000, Vraian, CA, USA).

## 4. Conclusions

In summary, we have successfully fabricated ultrathin, transparent and flexible capacitors on low-cost and flexible PEN substrates by ALD. H_2_O adsorption is necessary to make graphene electrodes on which high-quality ZrO_2_ dielectric films can be deposited. The capacitors show a relatively large capacitance of 10.3 fF/μm^2^ at 10 kHz with a low leakage current of 5.3 × 10^−6^ A/cm^2^ at 1 V, and can work at a bending radius of 10 mm without any deterioration of electrical performance in convex conditions. Ohmic conduction predominates when the field is lower than 0.2 MV/cm, while Schottky emission plays the main role when 0.2 < E < 0.7 MV/cm, and the SCLC mechanism is suitable when E > 0.8 MV/cm. The capacitors present an average optical transmittance of close to 70% in the visible range, opening possibilities for application in transparent and flexible integrated circuits.
